# On Medical Controversies

**Published:** 1807-11

**Authors:** 


### *To the Editors of the Medical and Physical Journal.*

GENTLEMEN,

YOUR correspondent Mr. Davies, has communicated in your Journal, a paper on Medical Controversies; and although the remarks in it are rather amusing than profound, I beg leave to draw the attention of your readers to the subject. It is well known that the *practical* opinions of medical men have been materially unsettled on the *subject* of gout, and of accidents from fire. It is well known, also, that the propagation of the cow-pox has given rise to many preposterous prejudices, and that, to allay these prejudices, Mr. RING has argued with great zeal and effect. These diseases have hitherto been considered as *practical subjects;* yet your correspondent, Mr. Davies, in your last number, attempts to cast a slur on your Journal for encouraging the investigation of them, and requests you in future to furnish him "more" with *practical subjects.* "It is to be be hoped, Sir," [I give his words and emphasis] "that your valuable miscellany will be furnished more with *practical* and less with *controversial* subjects, in future." What practice can it be that Mr. Davies so dictatorially calls for? I can assure Mr. Davies, that the diseases, which he considers only as controversial, are important objects of practice elsewhere. Even accidents from fire, [which are included in Mr. Davies's list of controversial subjects] so far require potent practice, that within the last half year I have witnessed no less than three *fatal* instances; and, what I consider, trifling treatment was alone pursued.

I am totally at a loss to understand Mr. Davies' ideas of controversy. Science at one time was a "warfare about words, and unfortunately this circumstance has brought the TERM controversy into disrepute. Is it the TERM only which offends Mr. Davies? In our science of course, as demonstration cannot be attained, there must exist a contrariety in evidence:—contrariety of authority, contrariety of experience, and, above all, diversity in habits of thinking. It is particularly the nature of the medical science to require comprehensive consideration; and without it, however ardent we may be in the pursuit of reason, we shall most probably go astray. Mr. Locke, on this subject writes very emphatically. There are many he observes, "who readily and sincerely follow reason, but for want of having that which one may call large, sound, round about sense, have not a full view of all that relates to the question, and may be of moment to decide it. We are all short sighted, and very often see but one side of a matter; our views are not extended to all that has a connection with it. From this defect I think no man is free. We see but in part, and we know but in part, and therefore it is no wonder we conclude not right from our *partial views*. This might instruct the proudest esteemer of his own parts how useful it is to talk and consult with others, even such as came short of him in capacity, quickness, or penetration." Now, comprehensive consideration necessarily *includes* controversy: for as we cannot form a solid judgement from *partial views* [in public or in private] we must either believe ALL OPINIONS, which would be incongruous, or CONTROVERT SOME. Controversy therefore is essential to the good foundation and improvement of Physic from the nature of the science. But we will condescend, as if proof was wanting, to apply the test of experience. For what reason did physic remain in the corrupted state in which it was left by the Arabians, and for centuries,

[Friend's History of Physic] but because error and ignorance were unsuspected and unexamined, and the faculties of the mind lay dormant? Compiler compiled from compiler; commentator commented upon commentator, and until authority was *controverted,* physicians continued in their ignorance. And does not controversy REMAIN equally important to the progressive improvement of physic? To discover truth—to clear up doubt—to dispel error; this is the definition of controversy. The object of investigation, is knowledge; but if, *in addition,* investigation is also pursued to eradicate prejudices; the investigation then goes by another name, and is called controversy. Such is the ambiguity of language.

The sources of error in the science of medicine are so numerous, that if controversy was confined to mere refutation, it could not with this restraint of its scope be too much cultivated. To mention only one of the many sources of error, which have yet been detected, I may instance the credulity of medical men in general. It is undeniable that many of our opinions are adopted, for which we cannot possibly give any account beyond mere faith in authority of professors. These professors had the same occasion for respect of, and confidence in, the authority of those from whom they had the advantage of reaping their knowledge. The farther we go back, the influence of authority gains ascendancy, and we come to the period before mentioned, when reason and experience were wholly suppressed, and when physicians did little more than register the prejudices of the times which preceded them. Habits of thinking too are hereditary; hence the many absurd doctrines which were conjured up in the ages of ignorance and superstition have been FAITHFULLY transmitted down to our times, and many of them still obtain credit. It is in this way that posterity is encumbered with the prejudices of their predecessors; and these prejudices are with the more difficulty detected, because *the writings of many are so disguised that it cannot be known what they give from experience, and what from conjecture.*

If error therefore was not daily springing up from wrong habits of thinking, controversy would be of the first consequence for the improvement of physic, to dissipate that error which is already entailed upon it.

I lately submitted to your readers two hasty papers; the one on the treatment of gout, the other on the treatment of burns and scalds; as these happen to be subjects which Mr. Davies wishes to be discontinued, because they are not practical, practical, I presumed to feel somewhat the weight of his censure. I must, however, be permitted to say, that if Mr. Davies has made up his mind to pursue the didactic style of instruction only, *non omne feret punctum,* till he mixes more of the utile and dulce. Mr. Davies is doing nothing in affirming it possible "that an opposite mode of treatment of the same disease may prove successful with patients of a different temperature," and then adding—a full stop! These affirmations have been printed again and again, and had an equal chance of producing conviction when supported by reason and experience, as when unsupported by either one or the other.

Upon the whole, Gentlemen, I think Mr. Davies has treated controversy in a supercilious and very superficial manner; and, if I may be allowed to retort his words, "it is to be hoped in future" he will confine HIMSELF to "*practical subjects.*"

I thought it too ridiculous to consider Mr. Davies's only objection to controversy. He compares [in some mysterious way] the reading a paper to the "ringing a peal," and he says, they have alike upon him, [if I understand the sentence] one effect, and that only on his "*Ears.*" It is needless to "RING" another peal in his ears. Perhaps Mr. Davies was rather unbecomingly reflecting on Mr. Ring's ingenious papers on the cow-pox, which have done much credit to your Journal. Doubtless in this case Mr. Davies considered himself *penning a good pun.*

I am, &c.

*September* 5, 1807. P. O.

